# Comparisons of Efficiency, Safety, and Hospital Costs of Four-Arm Robotic-Assisted Partial Nephrectomy (RAPN) Versus Three-Arm Technique: A Propensity Score–Matched Analysis

**DOI:** 10.3390/jcm14082739

**Published:** 2025-04-16

**Authors:** Yan Zhang, Fan Li, Wenhao Guo, Zongbiao Zhang, Heng Li, Wei Guan

**Affiliations:** Department of Urology, Institute of Urology, Tongji Hospital, Tongji Medical College, Huazhong University of Science and Technology, No. 1095 Jiefang Avenue, Wuhan 430030, China; zhangyanhust@tjh.tjmu.edu.cn (Y.Z.); m202376690@hust.edu.cn (W.G.); zzb070@tjh.tjmu.edu.cn (Z.Z.); lihengtjmu@tjh.tjmu.edu.cn (H.L.); deniskwan@hust.edu.cn (W.G.)

**Keywords:** robotic-assisted partial nephrectomy, port placement, hospital costs, propensity score matching

## Abstract

**Objectives**: The advent of robotic-assisted partial nephrectomy (RAPN) has marked a new era in minimally invasive surgery, establishing itself as a preferred method for managing renal cell carcinoma (RCC). However, even within the same center, variations in the use of robotic arms during RAPN have been reported. In this study, we aim to explore differences in efficiency, safety, and hospital costs between three-arm and four-arm RAPN. **Methods**: This retrospective study analyzed the clinical data of 91 patients who underwent RAPN in Tongji Hospital from January 2021 to December 2023. The patients were divided into two groups: 50 patients in the three-arm group (with the use of three robotic arms and two assistant ports) and 41 patients in the four-arm group (with the use of four robotic arms and one assistant port). Patients’ demographics and tumor characteristics, operative outcomes, and hospital costs were recorded. Propensity score matching (1:1) was performed on age, gender, body mass index, laterality, RENAL score, tumor stage, and pathological grade. We compared three-arm with four-arm RAPN groups based on operative outcomes, and hospital costs. **Results**: In total, 50 and 41 patients underwent three-arm and four-arm RAPN. All procedures were successfully executed without the need to convert to open surgery or radical nephrectomy. After matching, the four-arm configuration demonstrated a numerically longer total operative time compared with the three-arm approach (146.5 vs. 120.0 min, *p* = 0.068). Hospital costs in the four-arm group were significantly higher than those in the three-arm group (76,922.5 vs. 68,406.7 CNY, *p* = 0.006). **Conclusions**: Both the three-arm and four-arm robotic approaches demonstrated comparable safety and efficacy in RAPN procedures. Nevertheless, the four-arm approach is associated with elevated hospital costs. The preliminary findings suggest potential cost containment disadvantages for the four-arm technique in selected cases, though larger multicenter studies are essential.

## 1. Introduction

Renal cell carcinoma (RCC) accounts for 2–3% of adult malignancies worldwide and is in the top ten most common cancers diagnosed in both men and women [1]. Due to the advancements in imaging technology, 60% of patients are now diagnosed with RCC at an early stage [2]. For these patients, partial nephrectomy (PN) is recommended, to completely remove the lesion while maximally preserving renal parenchyma [3]. Although traditional open PN achieves a 95% 5-year cancer-specific survival rating [4], its 22–37% postoperative complication rate [4] has driven minimally invasive approaches into mainstream practice.

Laparoscopic PN emerged as a less invasive alternative. Compared with open PN, laparoscopic PN has less estimated blood loss, a shorter hospital stay, and a lower complication rate [5]. However, there are intrinsic limitations, such as rigid instruments and 2D visualization. Laparoscopic PN’s adoption is limited by the technical and ergonomic challenges during the crucial procedures of tumor excision and renal suturing (renorrhaphy) [6]. These constraints become the catalyst for robotic-assisted partial nephrectomy (RAPN), which now constitutes > 60% of minimally invasive PN procedures in high-volume centers [7].

The da Vinci surgical system addresses laparoscopic shortcomings through flexible instruments (7 degrees of freedom) and three-dimensional clear vision (10× magnified). Initial RAPN protocols utilized three robotic arms (camera + two working arms) [8], demonstrating a 55% reduction in acute renal function deterioration, and 1.3 days shorter hospital stay compared with laparoscopic PN [9]. The three-arm technique is now generally used in RAPN [10,11]. However, the introduction of the da Vinci S platform in 2006 enabled console surgeons to control an additional instrument. The fourth arm facilitates kidney retraction during hilar dissection, and thus theoretically reduces reliance on bedside assistants’ skill levels [12]. Since that time, several studies also described the RAPN technique utilizing four robotic arms (camera and three working arms) [13,14,15].

While four-arm RAPN has demonstrated 42 min operative time reductions in a single-center study [16], its broader clinical value remains contested due to cost concerns [17]. Crucially, existing comparisons suffer from selection bias in retrospective cohorts. Notably, no previous studies have employed propensity score matching to control for confounding factors.

In our retrospective analysis, we implement propensity score–matched analysis using seven covariates including age, gender, body mass index (BMI), laterality, RENAL score, tumor stage, and pathological grade, ensuring comparable baseline characteristics between three-arm and four-arm cohorts. This statistical approach minimizes selection bias inherent in observational studies, providing the most robust comparison to date. Secondly, we introduce a multidimensional evaluation framework encompassing seven clinically relevant domains: (1) Procedural efficiency (operative time, warm ischemia time), (2) hemostatic control (estimated blood loss, transfusion rate), (3) oncologic radicality (margin status), (4) convalescence metrics (post-operative analgesics use, and post-operative stay), (5) safety profiles (Clavien–Dindo complications), and (6) resource utilization (hospital costs). This multidimensional comparison seeks to inform evidence-based decisions regarding robotic platform utilization in PN.

## 2. Materials and Methods

### 2.1. Study Design

This retrospective study analyzed the clinical data of 91 patients submitted to RAPN in Tongji Hospital from January 2021 to December 2023. Patients with a T1-2 single renal tumor were included. Exclusion criteria included T3, multiple renal tumors, metastatic lesions, solitary kidney, and incomplete clinical data. Pre-operative evaluations were conducted for all patients, including CT and MRI.

The 91 patients were allocated into two groups: 50 patients comprised the 3-arm group, while 41 patients constituted the 4-arm group. The Da Vinci Si system (Intuitive Surgical, Sunnyvale, CA, USA) was used for RAPN in this research. All the operations were performed by one experienced surgeon Dr. Wei Guan (more than 300 cases). The Ethics Committee of Tongji Hospital approved this study design.

### 2.2. Surgical Technique

A gastric tube and catheter were inserted prior to the surgery. Following induction of general anesthesia, patients in both groups were positioned in the 70° flank position, with the affected side up. Pressure points were secured with special sponge pads, and bandages were used to stabilize the limbs. The operating table was mildly flexed to increase the space for ports. All surgeries were performed transperitoneally. The pneumoperitoneum was established, and the pressure was maintained at 12 mmHg. All ports were inserted under direct vision. The da Vinci robot was then docked to the robotic trocars.

**Figure 1:**
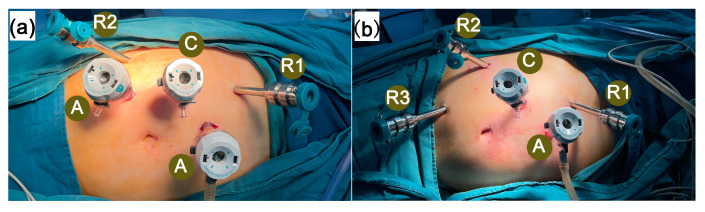
Port placement in robotic-assisted partial nephrectomy. (**a**) 3-arm port placement; (**b**) 4-arm port placement. C = camera port, R1 = first robotic port, R2 = second robotic port, R3 = third robotic port, A = assistant port.

In the 3-arm group, the ports were arranged as previously described [10]: one 12 mm camera port, two 8 mm robotic ports, and two 12 mm assistant ports. The camera port was positioned aligned with the lateral edge of the rectus abdominis, approximately 3 cm cephalad to the umbilicus. The 30-degree robotic camera was employed to facilitate the placement of other ports. The first robotic port was located cephalad to the camera port in the subcostal area, about 8 cm away. The second robotic port was located roughly 8 cm caudad to the camera port above the iliac crest, forming a 120° angle with the camera port and the first robotic port. The assistant ports consisted of two 12 mm Trocars: one was located vertically about 5 cm below the midpoint between the camera port and the first robotic port, and the other was located vertically 5 cm below the midpoint between the camera port and the second robotic port (Figure 1a). Slight adjustments of trocars were made according to tumor location.

The 4-arm group’s ports were arranged as follows [18]: one 12 mm camera port, three 8 mm robotic ports, and one 12 mm assistant port. The positions of the first and second robotic ports, the camera port, and the first assistant port were the same as those in the 3-arm group, with the third robotic port positioned caudad to the second robotic port, approximately 8 cm away (Figure 1b). Slight adjustments of trocars were made according to tumor location.

In the 3-arm group, RAPN was performed as previously described [10].

Left-sided RAPN procedure: An incision was made along the lateral peritoneal reflection adjacent to the descending colon, allowing access into the space between Gerota’s fascia and the descending colon. This dissection facilitated the medial mobilization of the descending colon. Near the lower pole of the kidney, Gerota’s fascia was incised in front of the psoas major to expose the left gonadal vein. The renal vein was proximally traced along the gonadal vein, while the ureter was identified laterally. The renal artery was identified posterior to the renal vein. In instances where the complex lumbar azygos vein structure was encountered or prominent first lumbar veins were present, the lumbar veins were tied off and cut to expose the renal artery. For patients with a high BMI, it is crucial to minimize disruption and transection of lymphatics to reduce the risk of post-operative lymphatic fistula [19]. Using pre-operative imaging or intra-operative ultrasound guidance (UST-5550T; Aloka, Tokyo, Japan), fat around the renal tumor was dissected and cleared to expose 2 cm of normal renal parenchyma, while fat on the tumor surface was not removed. After completely blocking the renal artery with Bulldog clips, the tumor was removed with cold scissors closely along the pseudo capsule of the tumor. Renorrhaphy was performed with 3-0 (for the deep vasculature) and 2-0 (for the outer renal parenchyma) V-loc absorbable sutures, and fastened with Hem-o-lok clips.

The right-sided RAPN procedure: The procedure started with an incision along the lateral peritoneal reflection adjacent to the ascending colon, entering the space between Gerota’s fascia and the ascending colon. By dissecting in this manner, we were able to medially mobilize the ascending colon, which in turn allowed the duodenum to be exposed in front of the inferior vena cava (IVC). Anterior Gerota’s fascia was incised to further expose the deeper anatomical structures. Loose connective tissues around the IVC were opened, allowing for the medial retraction of the descending part of the duodenum. The first major branch from the IVC’s right border was identified as the right renal vein. The remaining steps mirrored those of the left-sided procedure.

In the 4-arm group, an incision was made along the lateral peritoneal reflection adjacent to the descending/ascending colon, to medially mobilize the colon. The fourth arm was often used to retract tissues or hold the kidney. Near the lower pole of the kidney, Gerota’s fascia was incised in front of the psoas major. The lower pole of the kidney was lifted by the fourth robotic arm to identify the renal artery posterior to the renal vein. After completely blocking the renal artery, the tumor was removed and renorrhaphy was performed with 3-0 and 2-0 V-loc absorbable sutures.

Following that, the robot was undocked, and a perirenal drain was placed near the tumor bed. The specimen was bagged and retrieved via an extension of the camera port incision. Finally, all incisions were sutured.

### 2.3. Outcomes Measured and Statistical Analysis

Patients’ demographics and tumor characteristics such as age, gender, BMI, laterality, RENAL score, tumor stage, and pathological grade were collected. Operative outcomes such as preparative time, total operative time, warm ischemia time, estimated blood loss, blood transfusion, positive surgical margin, post-operative analgesics use, post-operative stay, and post-operative complications were reviewed. Hospital costs were also recorded. Tumor complexity was determined by the RENAL score, based on the five most reproducible features that characterize the anatomy of a solid renal mass on CT or MRI imaging [20]. The preparative time was defined as the duration from the initial incision to the full configuration of the robotic arms. The total operative time was defined as the time interval from skin incision to the completion of wound closure. Warm ischemia time was defined as the amount of time that the renal artery was blocked. Post-surgery complications were methodically categorized based on the Clavien–Dindo classification system [21].

We used a propensity score–matched analysis to control for treatment selection bias and confounding factors. Matching variables included age, gender, BMI, laterality, RENAL score, tumor stage, and pathological grade. On the basis of the propensity score, patients who underwent 3-arm RAPN were matched 1:1 without replacement to patients who underwent 4-arm RAPN using a nearest neighbor matching within a caliper set at 0.1. There was no missing data in our study. The balance of covariates before and after matching was assessed using standardized mean differences, with a value < 0.1 considered indicative of good balance.

All normality assessments were performed using the Shapiro–Wilk test. For data that followed a normal distribution, we reported the means and standard deviations. For data that did not follow a normal distribution, we reported the medians and inter-quartile ranges (IQR). Proportion was reported for categorical variables. We employed comparative tests (including Student’s *t*-test, Mann–Whitney U test, Chi-square test, or Fisher’s exact test) to evaluate differences between the two groups. Spearman correlation analysis assessed the correlation between variables. Data analysis was carried out using SPSS version 22.0 (IBM, Armonk, NY, USA), with *p* < 0.05 indicating statistical significance.

## 3. Results

### 3.1. Demographics and Tumor Characteristics

**Figure 2:**
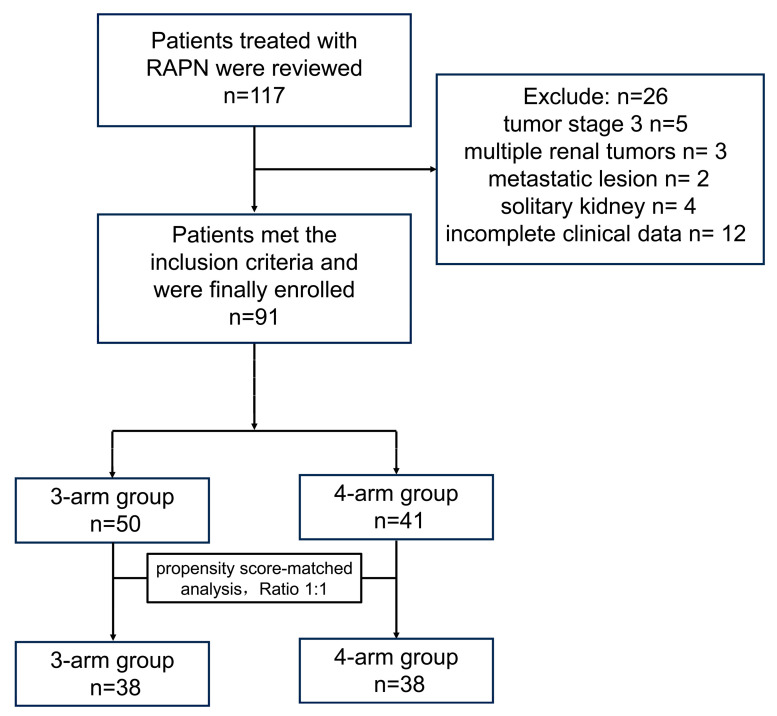
Patient selection flowchart. RAPN, robotic-assisted partial nephrectomy.

Figure 2 displays the patient selection process. Patients’ demographics and tumor characteristics are listed in Table 1. RAPN was performed on 91 patients in total, with all procedures successfully executed without the need to convert to open surgery or radical nephrectomy. All specimens were confirmed as RCC by pathology. The study participants were categorized into two distinct groups: 50 patients in the three-arm group (utilizing three robotic arms) and 41 patients in the four-arm group (utilizing four robotic arms). The median age for the two groups was 56.0 (*p* = 0.838). The male patient proportion showed a narrow gap between the three-arm group and four-arm group (72.7% vs. 70.7%, *p* = 0.894). The BMI was 24.7 ± 3.2 kg/m^2^ in the three-arm group, and 23.9 ± 2.6 kg/m^2^ in the four-arm group (*p* = 0.181). The three-arm cohort showed a higher prevalence of the left kidney with tumors, but this difference was not statistically significant (*p* = 0.141). There was no significant difference in tumor complexity between the two groups, as demonstrated by the median RENAL score (7.0 vs. 7.0, *p* = 0.761). A total of 80.6% of patients in the three-arm group and 75.6% of patients in the four-arm group were diagnosed at tumor stage T1a. In addition, RCC was commonly detected at pathological grade 2 among both groups, 32 out of 50 cases in the three-arm group (64%) and 19 out of 41 cases in the four-arm group (46.3%).

Using estimated propensity scores, 38 patients in the three-arm group matched 1:1 to patients in the four-arm group. Following the matching process, the differences in each parameter between the two groups were small or well matched. No significant differences were observed in demographics or tumor characteristics of patients between the two groups.

### 3.2. Operative Outcomes and Hospital Costs

Operative outcomes and hospital costs for the two cohorts are summarized in Table 2. The four-arm configuration demonstrated a numerically longer total operative time compared to the three-arm approach (146.5 vs. 120.0 min, *p* = 0.068). While this difference did not reach conventional statistical significance (α = 0.05), the observed difference (mean difference: 21 min, 95% CI: 0–43) suggests a clinically meaningful trend toward prolonged surgical duration. Comparing the four-arm and three-arm groups, preparative time (15.0 vs. 11.5 min, *p* = 0.080), warm ischemia time (30.0 vs. 28.0 min, *p* = 0.106), estimated blood loss (200.0 vs. 150.0 mL, *p* = 0.447), blood transfusion (13.2% vs. 13.2%, *p* = 1.000), positive surgical margin (2.6% vs. 2.6%, *p* = 1.000), post-operative analgesic use (3.0 vs. 3.0 days, *p* = 0.201), and post-operative stay (5.0 vs. 6.0 days, *p* = 0.694) demonstrated no statistically significant difference (*p* > 0.05). Notably, hospital costs were significantly higher in the four-arm group compared with those in the three-arm group (76,922.5 vs. 68,406.7, *p* = 0.006).

Details of the complications are listed in Table 3. There were no significant differences observed in the rate of post-operative complications between the three-arm group and the four-arm group (23.7% vs. 21.1%, *p* = 1.000). Most complications were slight (Clavien grade 1 or 2). One patient from each of the two groups developed a grade 3 complication of post-operative bleeding, requiring angioembolization. Two patients in the four-arm group developed deep vein thrombosis, requiring IVC filter. There were no grade 4 or 5 complications in this study.

**Figure 3:**
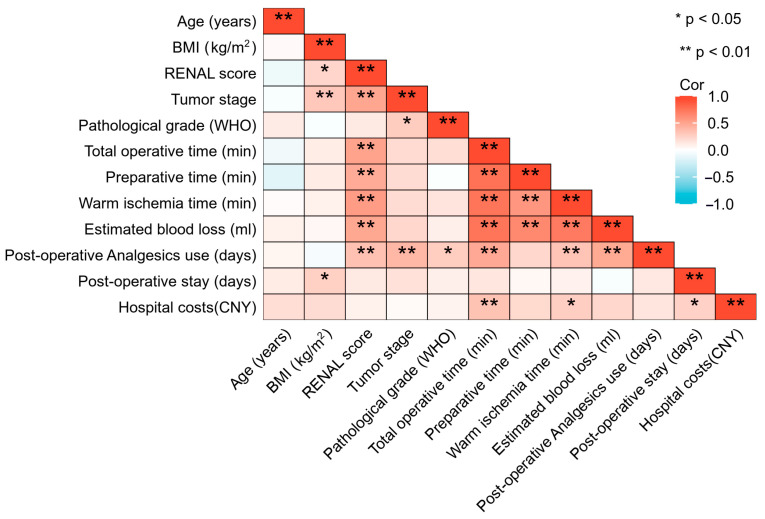
Spearman correlation analysis of RAPN patients’ parameters. BMI, body mass index; WHO, World Health Organization; CNY, Chinese Yuan.

For the entire cohort, Spearman correlation analysis revealed that hospital costs were correlated with total operative time, warm ischemia time and post-operative stay (Figure 3).

## 4. Discussion

At present, there is no definitive evidence to suggest significant differences between three-arm and four-arm RAPN in terms of operative and oncological outcomes and complications. Previous literature reports that some surgeons prefer to use the four-arm approach due to the greater maneuverability it provides, making the manipulation of surgical instruments easier. The fourth arm is particularly valued for its role in facilitating tissue retraction, exposure of the renal hilum, and renal artery and vein blocking, thereby reducing intra-operative dependence on surgical assistants [12,22]. However, other surgeons believe that three robotic arms are sufficient to complete the operation, and the three-arm approach can also ensure the efficiency of the surgery and achieve the desired surgical outcomes [17]. One study suggests that the three-arm technique has similar oncological and post-operative outcomes with less surgery costs [18].

RAPN is more costly than laparoscopic and open procedures [23]. In this study, the hospital costs for four-arm RAPN were significantly higher than those for three-arm RAPN, amounting to an additional 8515.8 Chinese Yuan (CNY). This increase is primarily due to the additional robotic arm, which necessitates more robotic surgical accessories, such as the ProGrasp^®^ forceps and sterile robotic arm drapes. Studies from other countries have reported that the hospital costs for four-arm RAPN are approximately 280–413 USD higher [17,18], which may be related to the diverse medical insurance systems. Additionally, prolonged total operative time increases anesthesia costs, which is another possible factor contributing to the rise in hospital costs. The four-arm approach for RAPN significantly increases costs without improving operative outcomes, making the three-arm approach more cost-effective and attractive.

While our study focused on moderate complexity tumors (median RENAL score = 7), emerging evidence suggests that four-arm RAPN may be particularly advantageous in complex renal tumors (e.g., hilar, endophytic, or multiple tumors), where precise resection and reconstruction are critical. The higher complexity of the renal tumor is associated with longer operative time, longer ischemia time, and more complications [24]. However, one study reporting on four-arm RAPN for complex tumors demonstrated a comparable operative time (197 vs. 215 min) and warm ischemia time (25 vs. 22 min) to the three-arm approach [14]. The four-arm approach is not universally cost-prohibitive, its role should be tailored to tumor complexity, and institutional resource availability. In settings with high complex tumor volumes, four-arm RAPN may represent a cost-neutral or even cost-saving strategy.

Intra-operative blood loss and warm ischemia time are important factors in evaluating the effectiveness of PN procedures [25]. The fourth arm is typically not utilized for tumor dissection and renorrhaphy and thus has no obvious impact on these parameters. This study found no significant differences between the two groups. Research by Schulze et al. shows similar outcomes, although more patients in the four-arm group required blood transfusion [18]. Due to the limited number of cases, larger studies are needed to evaluate the difference in blood transfusion rates. Another study suggests a modest reduction, approximately by 5 min, in warm ischemia time in the four-arm group, compared with the three-arm group [17]. However, differences in RENAL scores (6 vs. 7) could imply a selection bias, potentially influencing the outcomes [17].

Surgeon experience may influence operative outcomes. For instance, fellowship-trained surgeons demonstrated 18% shorter ischemia time (25.5 vs. 31.0 min) [26]. Increased surgical experience is associated with better operative outcomes, including fewer complications, shorter operative time, and shorter ischemia time [27,28]. Four-arm RAPN has a longer total operation time than three-arm RAPN (mean difference: 21 min, 95% CI: 0–43) in our study. However, even in the case of complex renal tumors, highly experienced surgeons could perform four-arm RAPN within a similar total operative time to three-arm RAPN (197 vs. 215 min) [8,14]. This suggests that technical proficiency mitigates four-arm workflow inefficiencies.

The experience of the surgeon and surgical assistant, as well as tumor characteristics, are important factors when considering the use of the fourth arm. Firstly, some surgeons are trained to use the four-arm technique and prefer it in subsequent surgeries. The assisting role of the fourth arm may be quite weak when the surgical assistant is highly skilled. Experienced surgeons may employ the fourth arm to compensate for less experienced assistants. Secondly, tumor characteristics, such as the inner-side location of the tumor, are associated with the non-use of the four-arm technique [29].

This study has some limitations. Firstly, it utilized the Da Vinci Si system, rather than the more contemporary Da Vinci Xi system, necessitating further studies to elucidate the benefits of the fourth arm across various platforms. As of December 2024, over 100 Si systems remain operational in Chinese hospitals, accounting for over 20% of the national da Vinci installed base. The average procurement cost of a new Xi system (over 20M CNY), makes wholesale replacement impractical for most institutions. For routine RAPN, the Si system demonstrates comparable peri-operative outcomes to the Xi system in case-matched cohort [30]. Thus, optimizing existing Si platform utilization is still meaningful. Secondly, the cases are from a single center with a relatively small patient number. More expansive, multi-center, prospective research is needed to obtain more definitive conclusions.

## 5. Conclusions

Both the three-arm and four-arm robotic approaches demonstrated comparable safety and efficacy in RAPN procedures. Nevertheless, the four-arm approach is associated with elevated hospital costs. The preliminary findings suggest potential cost containment disadvantages for the four-arm technique in selected cases, though larger multicenter studies are essential.

## Figures and Tables

**Table 1:** Demographics and tumor characteristics. IQR, inter-quartile range; SD, standard deviation; BMI, body mass index; WHO, World Health Organization.

	Before Propensity Score Maching			After Propensity Score Maching		
Variables	3-Arm Group (*n* = 50)	4-Arm Group (*n* = 41)	*p*	3-Arm Group (*n* = 38)	4-Arm Group (*n* = 38)	*p*
Age (years), median (IQR)	56.0 (49.8, 60.3)	56.0 (48.0, 63.0)	0.838	56.5 (49.3, 65.0)	55.5 (48.0, 60.5)	0.659
Male, *n* (%)	36 (72.0%)	29 (70.7%)	0.894	26 (68.4%)	27 (71.1%)	1.000
BMI (kg/m^2^), mean (SD)	24.7 (3.2)	23.9 (2.6)	0.181	24.1 (3.1)	24.1 (6.8)	0.995
Left, *n* (%)	24 (48.0%)	15 (36.6%)	0.141	17 (44.7%)	13 (34.2%)	0.348
RENAL score, median (IQR)	7.0 (6.0, 9.0)	7.0 (7.0, 9.0)	0.761	7.0 (6.0, 8.0)	7.0 (6.8, 9.0)	0.970
Tumor stage, *n* (%)			0.277			0.373
T1a	43 (86.0%)	31 (75.6%)		32 (84.2%)	28 (73.7%)	
T1b	3 (6.0%)	8 (19.5%)		2 (5.3%)	8 (21.0%)	
T2a	3 (6.0%)	2 (4.9%)		3 (7.9%)	2 (5.3%)	
T2b	1 (2.0%)	0 (%)		1 (2.6%)	0 (0%)	
Pathological grade (WHO), *n* (%)			0.375			0.986
1	14 (28.0%)	17 (41.5%)		13 (34.2%)	15 (39.5%)	
2	32 (64.0%)	19 (46.3%)		23 (60.5%)	18 (47.4%)	
3	3 (6.0%)	5 (12.2%)		2 (5.3%)	5 (13.1%)	
4	1 (2.0%)	0 (0%)		0 (%)	0 (%)	

**Table 2:** Operative outcomes and hospital costs (values are median (IQR) unless noted). IQR, inter-quartile range; CNY, Chinese Yuan.

Variables	3-Arm Group (*n* = 38)	4-Arm Group (*n* = 38)	*p*
Preparative time (min)	11.5 (10.0, 15.0)	15.0 (10.0, 20.0)	0.080
*Total operative time (min)*	*120.0 (100.0, 135.0)*	*146.5 (101.5, 177.8)*	*0.068*
Warm ischemia time (min)	28.0 (21.8, 33.3)	30.0 (25.8, 39.3)	0.106
Estimated blood loss (mL)	150.0 (100.0, 300.0)	200.0 (100.0, 362.5)	0.447
Blood transfusion, *n* (%)	5 (13.2%)	5 (13.2%)	1.000
Positive surgical margin, *n* (%)	1 (2.6%)	1 (2.6%)	1.000
Post-operative analgesic use (days)	3.0 (2.0, 3.0)	3.0 (3.0, 4.0)	0.201
Post-operative stay (days)	6.0 (5.0, 7.0)	5.0 (5.0, 7.0)	0.694
**Hospital costs (CNY)**	**68,406.7 (66,284.4, 74,542.7)**	**76,922.5 (68,474.3, 87,432.4)**	0.006 *

* **bold** *p* < 0.01, *Italic p* < 0.1.

**Table 3:** Post-operative complications (values are *n* (%)). SIRS, systemic inflammatory response syndrome.

Variables	3-Arm Group (*n* = 38)	4-Arm Group (*n* = 38)	*p*
Complications	9 (23.7%)	8 (21.1%)	1.000
Clavien Grade			0.673
1	6 (15.8%)	3 (7.9%)	
2	2 (5.3%)	2 (5.3%)	
3	1 (2.6%)	3 (7.9%)	
SIRS	2 (5.3%)	3 (7.9%)	1.000
Post-operative bleeding	1 (2.6%)	1 (2.6%)	1.000
Wound infection	2 (5.3%)	1 (2.6%)	1.000
Thrombosis	4 (10.5%)	3 (7.9%)	1.000

## Data Availability

The raw data supporting the conclusions of this article will be made available by the authors on request.

## Abbreviations

The following abbreviations are used in this manuscript:

RCC	renal cell carcinoma
RAPN	Robotic-assisted partial nephrectomy
CNY	Chinese Yuan
PN	partial nephrectomy
IVC	inferior vena cava
BMI	body mass index
IQR	inter-quartile range
WHO	World Health Organization
SD	standard deviation
SIRS	systemic inflammatory response syndrome

## References

[1] Fitzmaurice C., Allen C., Barber R.M., Barregard L., Bhutta Z.A., Brenner H., Dicker D.J., Chimed-Orchir O., Dandona R., Global Burden of Disease Cancer Collaboration (2017). Global, Regional, and National Cancer Incidence, Mortality, Years of Life Lost, Years Lived With Disability, and Disability-Adjusted Life-years for 32 Cancer Groups, 1990 to 2015: A Systematic Analysis for the Global Burden of Disease Study. JAMA Oncol..

[2] Ljungberg B., Campbell S.C., Choi H.Y., Jacqmin D., Lee J.E., Weikert S., Kiemeney L.A. (2011). The epidemiology of renal cell carcinoma. Eur. Urol..

[3] White V., Marco D.J.T., Bolton D., Davis I.D., Jefford M., Hill D., Prince H.M., Millar J.L., Winship I.M., Coory M. (2017). Trends in the surgical management of stage 1 renal cell carcinoma: Findings from a population-based study. BJU Int..

[4] Cozar J.M., Tallada M. (2008). Open partial nephrectomy in renal cancer: A feasible gold standard technique in all hospitals. Adv. Urol..

[5] You C., Du Y., Wang H., Peng L., Wei T., Zhang X., Li X., Wang A. (2020). Laparoscopic Versus Open Partial Nephrectomy: A Systemic Review and Meta-Analysis of Surgical, Oncological, and Functional Outcomes. Front. Oncol..

[6] Miller D.C., Hollingsworth J.M., Hafez K.S., Daignault S., Hollenbeck B.K. (2006). Partial nephrectomy for small renal masses: An emerging quality of care concern?. J. Urol..

[7] Alameddine M., Koru-Sengul T., Moore K.J., Miao F., Savio L.F., Nahar B., Prakash N.S., Venkatramani V., Jue J.S., Punnen S. (2019). Trends in Utilization of Robotic and Open Partial Nephrectomy for Management of cT1 Renal Masses. Eur. Urol. Focus..

[8] Gettman M.T., Blute M.L., Chow G.K., Neururer R., Bartsch G., Peschel R. (2004). Robotic-assisted laparoscopic partial nephrectomy: Technique and initial clinical experience with DaVinci robotic system. Urology.

[9] Bray G., Bahadori A., Mao D., Ranasinghe S., Tracey C. (2022). Benefits of Robotic Assisted vs. Traditional Laparoscopic Partial Nephrectomy: A Single Surgeon Comparative Study. J. Clin. Med..

[10] Kaouk J.H., Khalifeh A., Hillyer S., Haber G.P., Stein R.J., Autorino R. (2012). Robot-assisted laparoscopic partial nephrectomy: Step-by-step contemporary technique and surgical outcomes at a single high-volume institution. Eur. Urol..

[11] Tanaka K., Furukawa J., Shigemura K., Hinata N., Ishimura T., Muramaki M., Miyake H., Fujisawa M. (2015). Surgery-related outcomes and postoperative split renal function by scintigraphy evaluation in robot-assisted partial nephrectomy in complex renal tumors: An initial case series. J. Endourol..

[12] Rogers C.G., Laungani R., Bhandari A., Krane L.S., Eun D., Patel M.N., Boris R., Shrivastava A., Menon M. (2009). Maximizing console surgeon independence during robot-assisted renal surgery by using the Fourth Arm and TilePro. J. Endourol..

[13] Bhayani S.B. (2008). da Vinci robotic partial nephrectomy for renal cell carcinoma: An atlas of the four-arm technique. J. Robot. Surg..

[14] Gong Y., Du C., Josephson D.Y., Wilson T.G., Nelson R. (2010). Four-arm robotic partial nephrectomy for complex renal cell carcinoma. World J. Urol..

[15] Ener K., Canda A.E., Altinova S., Atmaca A.F., Alkan E., Asil E., Ozcan M.F., Akbulut Z., Balbay M.D. (2016). Robotic partial nephrectomy for clinical stage T1 tumors: Experience in 42 cases. Kaohsiung J. Med. Sci..

[16] El-Asmar J.M., Sebaaly R., Mailhac A., Bulbul M., Khauli R., Tamim H., El Hajj A. (2021). Use of Bariatric Ports in 4-Arm Robotic Partial Nephrectomy: A Comparative Study With the Standard 3-Arm Technique. Cureus.

[17] Johnson B.A., Crivelli J., Sorokin I., Gahan J., Cadeddu J.A. (2019). Surgical Outcomes of Three vs Four Arm Robotic Partial Nephrectomy: Is the Fourth Arm Necessary?. Urology.

[18] Schulze L., Dubeux V.T., Milfont J.C.A., Pecanha G., Ferrer P., Cavalcanti A.G. (2022). Analysis of surgical and histopathological results of robot-assisted partial nephrectomy with use of three or four robotic arms: An early series results. Int. Braz. J. Urol..

[19] Sforza S., Tellini R., Grosso A.A., Zaccaro C., Viola L., Di Maida F., Mari A., Carini M., Minervini A., Masieri L. (2020). Can we predict the development of symptomatic lymphocele following robot-assisted radical prostatectomy and lymph node dissection? Results from a tertiary referral Centre. Scand. J. Urol..

[20] Kutikov A., Uzzo R.G. (2009). The R.E.N.A.L. nephrometry score: A comprehensive standardized system for quantitating renal tumor size, location and depth. J. Urol..

[21] Dindo D., Demartines N., Clavien P.A. (2004). Classification of surgical complications: A new proposal with evaluation in a cohort of 6336 patients and results of a survey. Ann. Surg..

[22] Shiroki R., Fukami N., Fukaya K., Kusaka M., Natsume T., Ichihara T., Toyama H. (2016). Robot-assisted partial nephrectomy: Superiority over laparoscopic partial nephrectomy. Int. J. Urol..

[23] Alemozaffar M., Chang S.L., Kacker R., Sun M., DeWolf W.C., Wagner A.A. (2013). Comparing costs of robotic, laparoscopic, and open partial nephrectomy. J. Endourol..

[24] Huang Q., Gu L., Zhu J., Peng C., Du S., Liu Q., Chen J., Wang B., Fan Y., Gao Y. (2020). A three-dimensional, anatomy-based nephrometry score to guide nephron-sparing surgery for renal sinus tumors. Cancer.

[25] Tanagho Y.S., Figenshau R.S., Bhayani S.B. (2013). Technique, outcomes, and evolving role of extirpative laparoscopic and robotic surgery for renal cell carcinoma. Surg. Oncol. Clin. N. Am..

[26] Moriarty M.A., Nepple K.G., Tracy C.R., Strigenz M.E., Lee D.K., Brown J.A. (2016). Impact of Robotic Fellowship Experience on Perioperative Outcomes of Robotic-Assisted Laparoscopic Partial Nephrectomy. Curr. Urol..

[27] Larcher A., Muttin F., Peyronnet B., De Naeyer G., Khene Z.E., Dell’Oglio P., Ferreiro C., Schatteman P., Capitanio U., D’Hondt F. (2019). The Learning Curve for Robot-assisted Partial Nephrectomy: Impact of Surgical Experience on Perioperative Outcomes. Eur. Urol..

[28] Harke N.N., Kuczyk M.A., Huusmann S., Schiefelbein F., Schneller A., Schoen G., Wiesinger C., Pfuner J., Ubrig B., Gloger S. (2022). Impact of Surgical Experience Before Robot-assisted Partial Nephrectomy on Surgical Outcomes: A Multicenter Analysis of 2500 Patients. Eur. Urol. Open Sci..

[29] Kira S., Mitsui T., Sawada N., Nakagomi H., Ihara T., Takahashi N., Takeda M. (2020). Feasibility and necessity of the fourth arm of the da Vinci Si surgical system for robot-assisted partial nephrectomy. Int. J. Med. Robot..

[30] Abdel Raheem A., Sheikh A., Kim D.K., Alatawi A., Alabdulaali I., Han W.K., Choi Y.D., Rha K.H. (2017). Da Vinci Xi and Si platforms have equivalent perioperative outcomes during robot-assisted partial nephrectomy: Preliminary experience. J. Robot. Surg..

